# Recent Progress in 1,2-*cis* glycosylation for Glucan Synthesis

**DOI:** 10.3390/molecules28155644

**Published:** 2023-07-25

**Authors:** Akihiro Ishiwata, Katsunori Tanaka, Yukishige Ito, Hui Cai, Feiqing Ding

**Affiliations:** 1 RIKEN, Cluster for Pioneering Research, Saitama 351-0198, Japan; 2 Department of Chemical Science and Engineering, Tokyo Institute of Technology, Tokyo 152-8552, Japan; 3 Graduate School of Science, Osaka University, Osaka 560-0043, Japan; 4 School of Pharmaceutical Sciences (Shenzhen), Shenzhen Campus of Sun Yat-sen University, Shenzhen 518107, China

**Keywords:** α-glucan, stereoselective 1,2-*cis* glycosylation, α-glucosylation

## Abstract

Controlling the stereoselectivity of 1,2-*cis* glycosylation is one of the most challenging tasks in the chemical synthesis of glycans. There are various 1,2-*cis* glycosides in nature, such as α-glucoside and β-mannoside in glycoproteins, glycolipids, proteoglycans, microbial polysaccharides, and bioactive natural products. In the structure of polysaccharides such as α-glucan, 1,2-*cis* α-glucosides were found to be the major linkage between the glucopyranosides. Various regioisomeric linkages, 1→3, 1→4, and 1→6 for the backbone structure, and 1→2/3/4/6 for branching in the polysaccharide as well as in the oligosaccharides were identified. To achieve highly stereoselective 1,2-*cis* glycosylation, including α-glucosylation, a number of strategies using inter- and intra-molecular methodologies have been explored. Recently, Zn salt-mediated *cis* glycosylation has been developed and applied to the synthesis of various 1,2-*cis* linkages, such as α-glucoside and β-mannoside, via the 1,2-*cis* glycosylation pathway and β-galactoside 1,4/6-*cis* induction. Furthermore, the synthesis of various structures of α-glucans has been achieved using the recent progressive stereoselective 1,2-*cis* glycosylation reactions. In this review, recent advances in stereoselective 1,2-*cis* glycosylation, particularly focused on α-glucosylation, and their applications in the construction of linear and branched α-glucans are summarized.

## 1. Introduction

Stereoselective synthesis of 1,2-*cis* glycosides is one of the most challenging issues in the chemical synthesis of glycans [1,2,3,4,5,6,7]. Various 1,2-*cis* glycosides in nature have been found as α-glucoside and β-mannoside in glycoproteins, glycolipids, proteoglycans, microbial polysaccharides, and bioactive natural products. In the structure of polysaccharides such as α-glucan, 1,2-*cis* α-glucosides were found to be the major linkage between the glucopyranosides. Various regioisomeric linkages, 1→3, 1→4, and 1→6 for backbone structure, and 1→2/3/4/6 for branching in the polysaccharide as well as in the oligosaccharides were identified.

### α-D-glucans

α-D-glucan is a homopolysaccharide and a simple polymer of α-D-glucopyranoside (α-D-Glc*p*) [8,9]. D-Glucose, the component of the D-glucans, is photosynthesized in plants and widespread in nature and exists in its D-glucopyranose form in α-D-glucans [10]. The most common and linear example of α-D-glucan is (1→4)-α-D-glucan (amylose), which plays an essential role as an energy source for metabolism [11]. The chain length of amylose is known to be in the order of 500–6000 glucose units, depending on its botanical origin. Three crystalline forms of amylose, A-, B-, and C- (a mixture of A and B) granules [12], containing random and short helical segments, have been reported. Crystallized structures were found in the V form [13,14,15], and each segment composed of six glucose residues formed a left-handed, single-stranded helical structure [16]. Branched (1→4)-α-D-glucans are called amylopectin and glycogen, the analogues of starch for energy storage in plants and animals, fungi, and bacteria, respectively. The structures of amylopectin and glycogen are well known to be more compact than that of linear amylose. (1→4)-α-D-glucan is biologically synthesized by glucosyltransferase [17,18,19,20] and amylosucrase (sucrose-1,4-α-glucan glucosyltransferase [21,22,23,24] and (1→4)-α-D-glucan branching enzymes [25,26,27,28,29,30]).

**Figure 1:**
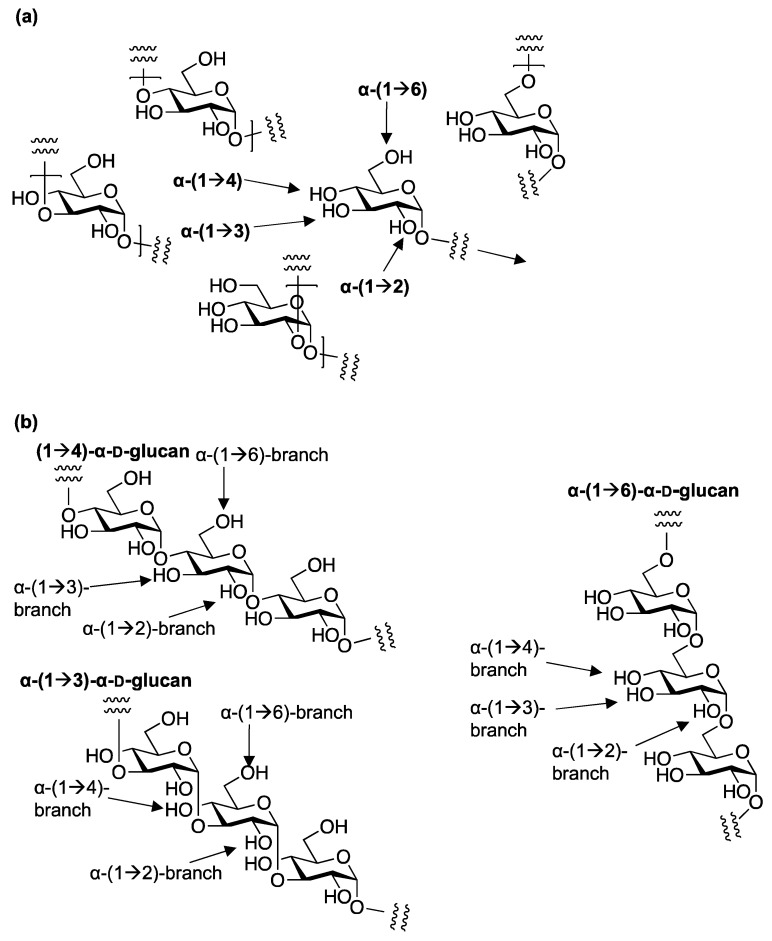
The linkages of α-D-glucans. (**a**) The linkages in the linear α-D-glucans; (**b**) the linkages of the branching in the various α-D-glucans.

α-D-glucans also have extremely complex structural diversity according to various regioisomers, making non-branched and branched α-D-glucans with (1→6)-, (1→4)-, (1→3)-, and (1→2)-glycosidic linkages and molecular masses according to the degree of polymerization (Figure 1). The α-D-glucans have been obtained from various species, listed in Table 1 [31,32,33,34,35,36].

Regioisomeric linear (1→6)-α-D-glucans (isomaltosides) were isolated from *Amillariella tabescens* and *Sarcodon aspratus* [37,38,39]. A dextran [40] obtained from lactic acid bacteria, such as *Lactobacillus*, *Leuconostoc*, *Weissella*, and *Streptococcus*, has a (1→6)-α-D-glucan backbone with up to 50% branching as α-(1→3), α-(1→4), or α-(1→2) linkages. Several glucosyl transferase (Gtf) enzymes synthesize dextrans with [41,42] and without branching [43,44,45,46,47,48]. The complex branched structures make the dextrans effective energy storage molecules that release D-glucose slowly via enzymatic hydrolysis [49,50,51,52,53].

Linear (1→3)-α-D-glucan (pseudonigan) was identified from *Aspergillus niger* [48] as a storage polysaccharide [54]. To the best of our knowledge, a linear (1→2)-α-D-glucan has not yet been identified. The (1→3)-α-D-glucans are major components of the cell wall of filamentous fungi [55,56,57] and dimorphic yeasts [58,59,60,61] and are synthesized via the primer for (1→3)-α-D-glucans by intracellular amylases. The structural analysis of (1→3)-α-D-glucan was reported and it was mentioned that three crystalline forms I–III of (1→3)-α-D-glucan were detected and interconverted via dehydration and hydration reactions [32,62]. Various biological functions of (1→3)-α-D-glucan were investigated such as immunological activity via Toll-like receptor 4 (TLR4) [63,64,65], which has been shown in the case of (1→4)-α-D-glucans as well as β-D-glucans [66].

More complex branching structures have been discovered in various linear glucans [33,67,68]. From dextran, NRRL B1397, an α-D-Glc-(1→2)-α-D-Glc-(1→6)-D-Glc structure [69,70,71,72,73] was identified and the D-Glc-(1→2)-branching moiety was found to be an α-glucoside to tricholomal (1→4)-α-D-glucan [74].

The most common and linear example of a stereoisomeric β-D-glucans is cellulose, composed of β-D-Glc*p*, which plays a fundamental role as a structural component of the cell wall [75,76]. As physiologically active biological response modifiers (BRMs), the structure of glucans and the biological activity relationship of β-D-glucans have been reported to be adjuvants in bacterial, viral, or protozoan infections, and potent antitumor drugs, depending on the molecular weight, degree of branching, conformation, and intermolecular associations of glucans [76,77,78,79,80,81]. In the case of the synthesis of β-D-glucans, a common methodology such as stereoselective β-D-glucopyranosylation via the effect of neighboring group participation from the 2-*O*-acyl group can be effectively used [82,83,84].

**Table 1:** Various α-D-glucans in nature.

Linkage		Name	Source	Ref.
Linear	Side Chain			
(1→4)-α	-	Amylose	*Mycobacterium tuberculosis*	[85,86]
(1→4)-α	-	Amylose	*Streptomyces venezuelae*	[87]
(1→4)-α	-	Amylose	*Fusicoccum amygdale*	[88]
(1→4)-α	-	Amylose	*Agaricus blazei*	[89]
(1→4)-α	-	Amylose	*Pleurotus ostreatus*	[84]
(1→4)-α	-	Starch	*Rice bran*	[90]
(1→4)-α	(1→6)-α	Glycogen	*Saccharomyces cerevisiae*	[91]
(1→4)-α	(1→6)-α	Glycogen	*Agaricus bisporus*	[92]
(1→4)-α	(1→6)-α	Glycogen	*Cordyceps sinensis*	[93]
(1→4)-α	(1→6)-α	Glycogen	*Coprinus comatus*	[94]
(1→4)-α	(1→6)-α	Glycogen	*Flammulina velutipes*	[95]
(1→4)-α	(1→6)-α	Glycogen	*Gastrodia elata Bl*	[96]
(1→4)-α	(1→6)-α	Glycogen	*Lonicera japonica Thunb*	[97]
(1→4)-α	(1→6)-α	Glycogen	*Actinidia chinensis*	[98]
(1→4)-α	(1→2); (1→6)-α	Glycogen	*Tricholoma matsutake*	[71]
(1→4)(1→6)-α	-	Reuteran	*Lactobacillus reuteri*	[99]
(1→4)(1→6)-α	-	Pullulan	*Aureobasidium pullulans* *Cyttaria harioti* *Tremella mesenterica*	[100]
(1→4)(1→6)-α	-	Pullulan	*Tremella mesenterica*	[101]
(1→3)-α	-	Pseudonigeran	*Aspergillus flavipes* *Aspergillus flavus* *Aspergillus fumigatus* *Aspergillus ochraceus*	[102]
(1→3)-α	-	-	*Aspergillus fumigatus*	[103,104,105,106]
(1→3)-α	-	Pseudonigeran	*Aspergillus nidulans*	[107,108,109]
(1→3)-α	-	-	*Aspergillus niger*	[110,111]
(1→3)-α	-	Pseudonigeran	*Aspergillus niger NNRL 326*	[111]
(1→3)-α	-	-	*Aspergillus wentii*	[112]
(1→3)-α	-	Pseudonigeran	*Blastomyces dermatiditis (yeast form)*	[113,114]
(1→3)-α	-	Pseudonigeran	*Eupenicillium crustaceum*	[111]
(1→3)-α	-	Pseudonigeran	*Fusarium oxysporum*	[111]
(1→3)-α	-	Pseudonigeran	*Fusicoccum amygdale*	[88]
(1→3)-α	-	Pseudonigeran	*Histoplasma capsulatum*	[115]
(1→3)-α	-	Pseudonigeran	*Histoplasma farciminosum*	[116]
(1→3)-α	-	Pseudonigeran	*Paracoccidioides brasiliensis*	[117]
(1→3)-α	-	Pseudonigeran	*Penicillium brevi-compactum* *Penicillium decumbens*	[102]
(1→3)-α	-	Pseudonigeran	*Penicillium expansum*	[118]
(1→3)-α	-	Pseudonigeran	*Penicillium chrysogenum*	[119]
(1→3)-α	-	Pseudonigeran	*Poria cocos*	[120]
(1→3)-α	-	Pseudonigeran	*Agrocybe cylindracea*	[121]
(1→3)-α	-	-	*Amanita muscaria*	[122]
(1→3)-α	-	Pseudonigeran	*Armillaria mellea*	[123]
(1→3)-α	-	Pseudonigeran	*Cryptococcus albidus*	[124]
(1→3)-α	-	Pseudonigeran	*Cryptococcus terreus*	[124]
(1→3)-α	-	Pseudonigeran	*Ganoderma lucidum*	[125]
(1→3)-α	-	Pseudonigeran	*Ganoderma tsugae*	[126]
(1→3)-α	-	-	*Laetiporus sulphureus*	[127]
(1→3)-α	-	Pseudonigeran	*Lentinus edodes*	[128]
(1→3)-α	-	Pseudonigeran	*Piptoporus betulinus*	[127]
(1→3)-α	-	Pseudonigeran	*Pleurotus ostreatus*	[36]
(1→3)-α	-	Pseudonigeran	*Pleurotus eryngii*	[88]
(1→3)-α	-	Pseudonigeran	*Polyporus tumulosus*	[129]
(1→3)-α	-	Pseudonigeran	*Schizophyllum commune*	[130]
(1→3)-α	-	Pseudonigeran	*Tremella mesenterica*	[101]
(1→3)-α	(1→6)-α	Mutan	*Lactobacillus reuteri* *Streptococcus mutans* *Streptococcus salivarius* *Streptococcus sownei*	[131,132]
(1→3)(1→4)-α	-	Nigeran	*Aspergillus niger var.awamori* *Aspergillus niger var.unknowy* *some Aspergillus species*	[133]
(1→3)(1→4)-α	-	-	*Aspergillus wentii*	[112]
(1→3)(1→4)-α	-	-	*Cladosporium herbarum*	[134]
(1→3)(1→4)-α	-	Elsinan	*Elsinoe leucospila*	[135]
(1→3)(1→4)-α	-	-	*Neurospora crassa*	[136]
(1→3)(1→4)-α	-	Nigeran	*Few other Penicillium species*	[137]
(1→3)(1→4)-α	-	-	*Schizosaccharomyces pombe*	[124]
(1→3)(1→4)-α	-	Nigeran	*Armillaria mellea*	[123]
(1→3)(1→4)-α	-	-	*Coriolus versicolor*	[138]
(1→3)(1→4)-α	-	Pseudonigeran	*Cryptococcus neoformans*	[139]
(1→3)(1→4)-α	-	Pseudonigeran	*Laetiporus sulphureus*	[127]
(1→3)(1→4)-α	-	Pseudonigeran	*Lentinus edodes*	[128]
(1→3)(1→4)-α	-	Isolichenin	*Cetraria richardsonii*	[140]
(1→3)(1→4)-α	-	Isolichenin	*Cetraria islandica*	[140]
(1→3)(1→4)-α	-	Isolichenin	*Letharia vulpine*	[140]
(1→3)(1→4)-α	-	Everniin	*Evernia prunastri*	[141,142]
(1→3)(1→4)-α	-	Nigeran	*Parmelia carperata* *Parmelia cetrarioides* *Ramalina species,* *Cladonia species*	[140]
(1→3)(1→4)-α	-	Isolichenin	*Alectoria sarmentosa* *Alectoria sulcate* *Cetraria species* *Usnea species* *Parmelia species*	[141,142]
(1→3)(1→6)-α	(1→3)-α	Alternan	*Leuconostoc mesenteroides* *Streptococcus salivarius*	[131,132]
(1→3)(1→6)-α	-	-	*Termitomyces eurhizus*	[45]
(1→3)(1→4)(1→6)-α	-	Acroscyphan	*Acroscyphus sphaerophoroides*	[141,142]
(1→6)-α	-	-	*Coriolus versicolor*	[138]
(1→6)-α	-	-	*Sarcodon aspratus*	[143]
(1→6)-α	-	-	*Termitomyces eurhizus*	[45]
(1→6)-α	-	Starch	*Banana*	[144]
(1→6)-α	-	Starch	*Dimocarpus longan Lour cv Shixia*	[145]
(1→6)-α	-	Starch	*Pueraria lobata (willed) ohwi*	[146]
(1→6)-α	-	Starch	*Ipomea batatus*	[147]
(1→6)-α ^1^	-	-	*Chlorella vulgaris*	[148]
(1→6)-α	(1→3)-α	-	*Lobelia chinensis*	[149]
(1→6)-α	(1→2); (1→3); (1→4)-α	Dextran	*Lactobacillus species* *Leuconostoc dextranicum* *Leuconostoc mesenteroides* *Streptococcus mutans* *Weissella species*	[131,132]
(1→2)-α		-	*-*	-

^1^ sulfated glucan.

## 2. 1,2-*cis* glycosylation

Stereoselective *O*-glycosylation is a key step in the assembly of biologically relevant oligosaccharides. The target oligosaccharide contains 1,2-*cis*- or 1,2-*trans*-configurated *O*-glycosidic linkages to the C-2–O bond of the non-reducing side residue of the glycoside. The 1,2-*cis* linkages, such as α-glucopyranoside, α-galactopyranoside, β-mannopyranoside, β-rhamnopyranoside, and other glycosides, are found in natural glycans, including glycoconjugate such as glycoproteins, glycolipids, proteoglycans, microbial polysaccharides, and glycosylated natural products. Controlling the stereoselectivity in the formation of 1,2-*cis* glycosides is extremely challenging in synthetic chemistry, as in the case of α-gluco (2-equatorial)- and β-manno (2-axial)-type glycoside formations, although the method for the 1,2-*trans* isomers was developed by using the effect of neighboring group participation from theC-2 acyl group as the first choice of the chemist. Various methods using inter- [150,151,152,153,154,155] and intra- [156,157,158] molecular procedures have been developed for the stereoselective synthesis of 1,2-*cis* glycosides [153,159], depending on the acceptor molecules [160,161], and further developments have been reported in recent years [162,163,164,165].

**Figure 2:**
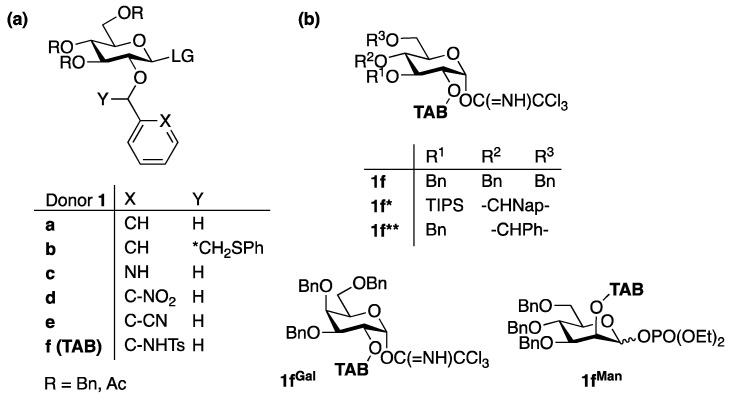
2-*O*-ether-protected glycosyl donors. (**a**) The 2-*O*-ether-protected glycosyl donors for stereoselective glycosylation; (**b**) TAB-protected donors for bimodal glycosylations.

The 2-*O*-ether-protected glycosyl donors predominantly afford the axial glycosides via stereoelectronic effects [166,167,168,169,170,171,172,173] (Figure 2). Using this methodology, 1,2-*cis* gluco-type pyranosides were selectively obtained. However, the selectivity is not predictable, mainly because of the many controversial results reported from a variety of examinations using many types of donors suitably optimized to the demand of their targets. Based on basic observations, the solvent effect [174,175,176,177,178,179,180], the concentration effect [181,182,183,184,185], and other factors [186,187,188], including a very recent approach using an S_N_2-predicting, leaving group enhanced by a coordinating acceptor [189,190], were also accepted as factors for the stereoselectivity of glycosylation. This review focuses on two effective and stereoselective methods for glucan synthesis: the use of C2-*o*-tosylamide (TsNH)-benzyl (TAB) ether for bimodal glycosylation [191,192,193] and ZnI_2_-mediated 1,2-*cis* glycosylation [194].

### 2.1. Bimodal Glycosylation Approach

#### 2.1.1. Bimodal Glycosylation Approach for 1,2-*cis* α-glucosylation

**Scheme 1:**
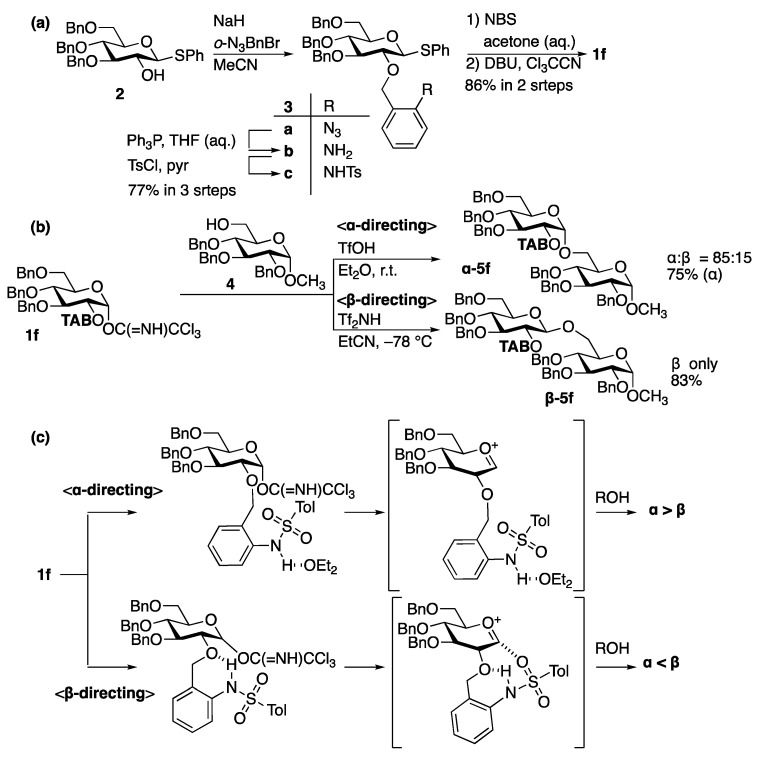
Bimodal glycosylation of TAB-protected glucosyl donor **1f**. (**a**) Synthesis of TAB-protected donor **1f**; (**b**) bimodal glycosylation condition using single TAB-protected donor **1f** by changing the reaction conditions; (**c**) proposed pathway of stereoselection for both 1,2-*cis* and 1,2-*trans* glucosylation.

Because of the structural diversity of glucans, a unified strategy for the assembly of pure glucans is yet to be developed. For the stereocontrolled synthesis of both α- and β-glycosides, a general strategy that applies to the construction of all types of glucans by exploiting a bimodal [195,196,197,198,199] glucosyl donor equipped with C2-*o*-TAB ether [200,201,202,203,204] by the simple switching of the reaction conditions was developed in our laboratory [191,192] (Scheme 1). The synthesis of the glycosyl trichloroacetimidate donor with C2-*O*-TAB ether was carried out through a five-step transformation from the C2-OH of the thioglycoside derivative via C2-*O*-ether formation with *o*-azidobenzyl bromide [205,206] and NaH, reduction in the azide moiety by triphenylphosphine, and tosylation of the resultant amine. This was followed by the hydrolysis of the thioglycoside and subsequent treatment with trichloroacetonitrile in the presence of 1,8-diazabicyclo[5.4.0]undec-7-ene (DBU) (Scheme 1a).

The selective formation of β-glucosides was achieved when the activation of trichloroacetimidate was carried out by bis(trifluoromethanesulfonyl)imide (Tf_2_NH) [207,208,209,210] in propionitrile (EtCN) at low temperatures (−40 to −78 °C) (β-directing conditions). Using the same glucosyl donor, an alternative activation by triflic acid (TfOH) in Et_2_O under diluted conditions at room temperature predominantly provided α-glucosides as the major product (α-directing conditions) (Scheme 1b). After glucosylation, the selective liberation of the 3-, 4-, or 6-OH functionality in the presence of the TAB group at the C2 position and deprotection of the TAB group to liberate the 2-hydroxy group allowed for further glycosylations. The versatility of the bimodal glucosylation method was demonstrated by effectively assembling fragments of natural and non-natural glucans [191].

When the PhSO_2_ group of an equatorially oriented TAB group at the 2-*O*-position interacts with the glycosyl cation through neighboring group participation in the presence of Tf_2_NH in EtCN, β-glycosides are predominantly formed (Scheme 1c). The stereodirecting effects of the TAB group have been explained by the contribution of hydrogen bonding between tosylamide and benzylic oxygen, forming a quasi-bicyclic form, such as the 2-phthalimide (NPhth) group as a 1,2-*trans* directing group [211,212,213]. The activation of the donor moiety to initiate the formation of the oxocarbenium ion results in subsequent NGP by the sulfonamide oxygen to provide β-glycosides. Contrary to 1,2-*trans*-glycosylation, in ether solvents, the disruption of the intramolecular hydrogen bonding may result in the interaction with the incoming acceptor via intermolecular hydrogen bonding, controlling the 1,2-*cis* attack to afford α-glucosides selectively. Reactions using the perbenzylglucosyl trichloroacetimidate donor without the NHTs group in the presence of Tf_2_NH in EtCN provided the corresponding glycosides with diminished stereoselectivity (α/β = 11/ 89 compared with β-only for **6a**), whereas the stereoselectivity was similar (α/β = 83/17) to **6a** (84/16) in the presence of TfOH in Et_2_O [191]. These results also support the proposed mechanism.

#### 2.1.2. Bimodal Glycosyl Donor Approach for Application to 1,2-*cis* α-galactosylation and 1,2-*cis*-β-mannosylation

**Scheme 2:**
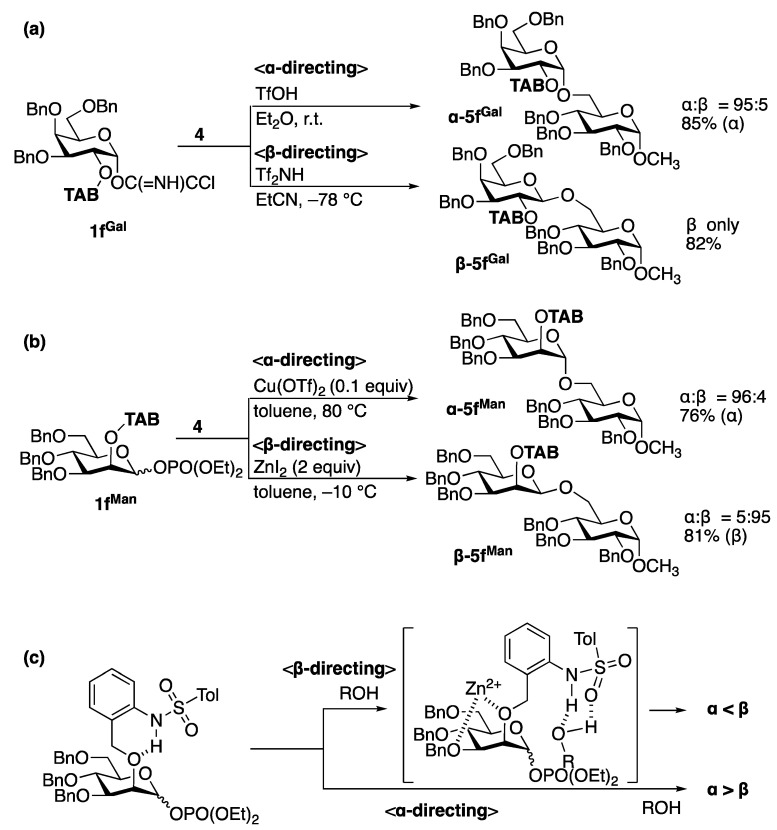
Bimodal glycosylation of TAB-protected galactosyl and mannosyl donors (**1f^Gal^** and **1f^Man^**). (**a**) Bimodal galactosylation conditions; (**b**) bimodal mannosylation conditions; (**c**) proposed pathway of stereocontrol for mannosylation of **1f^Man^**.

The bimodal α- and β-glycosylations were simply applied to the stereoselective synthesis of both α- and β-galactosides using a bimodal galactosyl donor with C2-*O*-TAB ether by the simple switching of the α- and β-directing reaction conditions, respectively, optimized for glucosylation [191]. The galactosyl donor has equatorial C2-*O*-TAB, which should similarly induce α- and β-selectivity as the glucosyl donor (Scheme 2a).

As in the case of bimodal α- and β-glucosylation, it was found that the hydrogen bond donating ability of the TsNH group of the 2-*O*-TAB group caused an interaction with the incoming alcohol (ROH), thereby leading to 1,2-*cis*-selective glycosylation, as mentioned before. Next, 2-*O*-TAB was used for 1,2-*cis*-selective mannosylation [214]. In addition, by changing the reaction conditions that disrupt the intermolecular hydrogen bonding, the selective formation of the 1,2-*trans*-α-glycoside is possible (Scheme 2b). Although the construction of α-mannosyl linkages can be achieved by neighboring group participation or through the exploitation of stereoelectronic effects, the β-linkage of mannoside is challenging to construct stereoselectively [215]. Well-established methodologies for β-mannoside synthesis include direct glycosylation with 4,6-*O*-benzylidene protected donors [216,217,218,219,220,221,222] and indirect methodologies, including intramolecular aglycon delivery (IAD) [156,223,224,225,226,227,228,229], intermolecular H-bond-mediated aglycone delivery [230], stereochemical inversion of β-gluco or β-galacto glycosides [231,232,233,234,235], and anomeric *O*-alkylations [236].

An overstoichiometric amount of the Zn^2+^ salt (2 equiv.) is required for β-mannosylation using a mannosyl donor with an imidate or phosphite as the leaving groups. Under these conditions, oxygen atoms at the 2- and 3-position coordinate with Zn^2+^, cleaving the intramolecular hydrogen bonding [237,238] (Scheme 2c). Afterward, the liberated NH group will be able to interact with an incoming nucleophile in an intermolecular fashion, reversing the stereocontrolling effect of the TAB group. The use of Cu(OTf)_2_ in toluene, especially at elevated temperatures with the same phosphite donor, afforded the α-isomer predominantly. The application of this bimodal mannosyl donor to the synthesis of all possible stereoisomers of trisaccharide D-Man-(1→2)-D-Man-(1→6)-D-Glc [239,240,241,242,243] was achieved.

### 2.2. ZnI_2_-mediated Stereoselective Glycosylation Approach

#### 2.2.1. ZnI_2_-mediated 1,2-*cis* α-glucosylation

**Figure 3:**
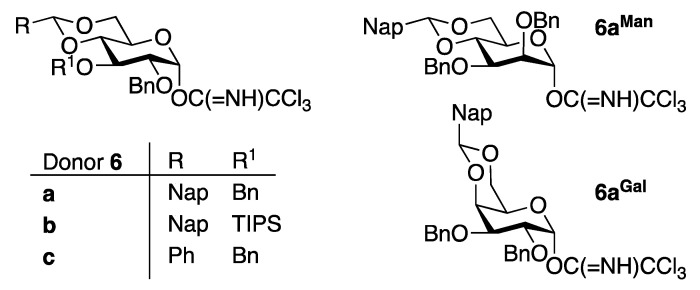
Donors for ZnI_2_-mediated 1,2-*cis* glycosylations.

**Scheme 3:**
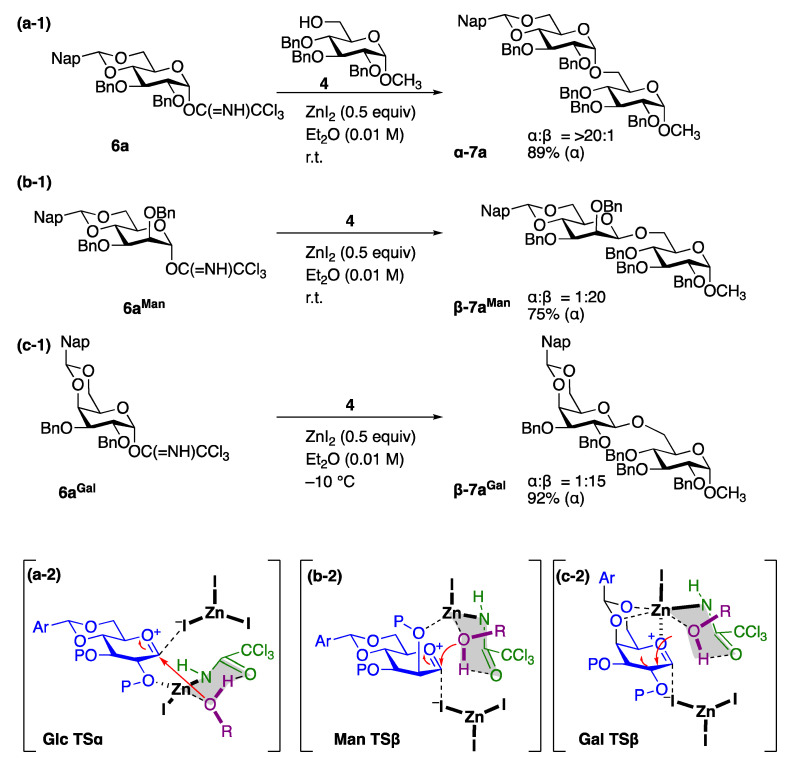
ZnI_2_-mediated 1,2-*cis* glycosylations. (**a-1**) Stereoselective 1,2-*cis* α-glucosylation and (**a-2**) TS structure for 1,2-*cis* α-glucosylation; (**b-1**) stereoselective 1,2-*cis* β-mannosylation and (**b-2**) TS structure for 1,2-*cis* α-glucosylation; (**c-1**) stereoselective *cis* β-galactosylation and (**c-2**) TS structure for 1,2-*cis* α-glucosylation. TS structures were proposed by DFT calculations. For Ar, P, and R, CH_3_-, CH_3_-, and CH_3_CH_2_- were used for the calculation instead of Nap, Bn, and an acceptor molecule, respectively.

As shown in the case of bimodal α- and β-mannosylations using the 2-*O*-TAB group that interacted with/without the acceptor (ROH), the effect of Zn^2+^ salt (2 equiv.) was revealed for β-mannosylation, using the mannosyl donor with imidate or phosphite as the leaving group. It has been observed that the Zn^2+^ cation not only activates the donor leaving group but also coordinates with oxygens at the 2- and 3- positions to induce the effective interaction of TAB with an incoming nucleophile during 1,2-*cis*-β-mannosylation [214]. Combined with the enhancement of the fixed conformation of the pyranose ring by the 4,6-*O*-cyclic protection reported by Crich [244,245], a simple ZnI_2_-mediated procedure involving activation and direction to control the stereoselectivity for glucosylation has been developed as a novel general synthetic strategy for the construction of α-glucoside as one of the most abundant 1,2-*cis*-glycosidic bonds in nature [194]. To the best of our knowledge, the effective use of ZnI_2_ for 1,2-*cis* glycosylation using a simple trichloroacetimidate donor has not been reported until recently. Using various acceptors, ZnI_2_-mediated α-glucosylation was demonstrated using 4,6-*O*-naphthylidene (NapCH<)-protected donors (**6a**) to demonstrate its versatility and effectiveness (Figure 3, Scheme 3(a-1)). The modular synthesis of various α-glucans with both linear and branched backbone structures using this simple approach was successfully achieved, as described in Section 3.2.1.

In addition to the experimental investigations and theoretical calculations, the ZnI_2_-mediated 1,2-*cis* glycosylation was analyzed (Scheme 3(a-2)) [194]. Theoretically, ZnI_2_ activates the anomeric leaving groups on the donor molecule as Lewis acids and enhances glycosyl iodide formation. Subsequent activation of glycosyl iodide by another ZnI_2_ leads to an intermediate that is also coordinated with the first ZnI_2_, which effectively coordinates with both hydroxyl groups on the acceptor, forming a six-membered structure with a trichloroacetimidate ion and the O-2 of the donor. Subsequent stereocontrolled nucleophilic attacks from the same side to the O-2 of the donor afford a 1,2-*cis* linkage, which then dissociates to the desired products.

#### 2.2.2. ZnI_2_-mediated 1,2-*cis* β-mannosylation, and *cis* β-galactosylation

As we have successfully developed a ZnI_2_-directed general strategy for 1,2-*cis* α-glucosylation using a 4,6-*O*-naphthylidene and 2-*O*-benzyl (Bn)-protected glucosyl donors with excellent stereoselectivity [194], the ZnI_2_-mediated 1,2-*cis* glycosylation strategy has been applied to other linkages, such as 1,2-*cis* β-mannosides [217] and 1,2-*cis* α-galactosides [246]. In recent years, various methods have been developed [247,248,249] for stereoselective glycosylation to obtain more difficult 1,2-*cis* linkages with equatorial glycosides found in the core structure of the *N*-glycans [250,251,252,253,254,255,256,257]. The ZnI_2_-directed strategy can be extended to the 4,6-*O*-tether and 2-*O*-benzyl-protected mannosyl trichloroacetimidate donors [217], promising an alternative β-mannosylation methodology via a similar 1,2-*cis* stereoselectivity to 1,2-*cis* α-glucosylation (Figure 3, Scheme 3(b-1)). ZnI_2_-promoted mannosylation has also been used to synthesize the core structure of *N*-glycan effectively. The ZnI_2_ coordination with both a hydroxyl group on the acceptor and the O-2 of the donor after glycosyl iodide formation, followed by anomerization from the β- to α-isomer and the subsequent activation of α-glycosyl iodide by the second ZnI_2_, afforded a 1,2-*cis* β-mannosidic linkage [217] (Scheme 3(b-2)).

In contrast, glycosylation with 4,6-*O*-naphthylidene and 2-*O*-benzyl-protected galactosyl trichloroacetimidate donor (**6a^Gal^**) under ZnI_2_ activation conditions resulted in 1,2-*trans* β-galactosylation [246] (Figure 3, Scheme 3(c-1)). Based on the experimental and theoretical investigations, β-galactosylation should be promoted by the dual roles of the proposed zinc cations as the activator and mediator of the structural restriction-enhanced *cis* stereodirecting intermolecular interaction, unexpectedly from the 4- or 6-position of the 4,6-*O*-naphthylidene-protected galactosyl donor, and not from the 2-position, as in the 1,2-*cis* cases of glucosylation and mannosylation (Scheme 3(c-2)).

## 3. Recent Progress on the Synthesis of α-glucans

### 3.1. Application of the Bimodal Glycosylation Approach for Stereoselective 1,2-cis α-glucosylation toward the Synthesis of α-glucans

#### 3.1.1. Bimodal Glycosylation Approach for the Synthesis of Linear α-glucans

**Scheme 4:**
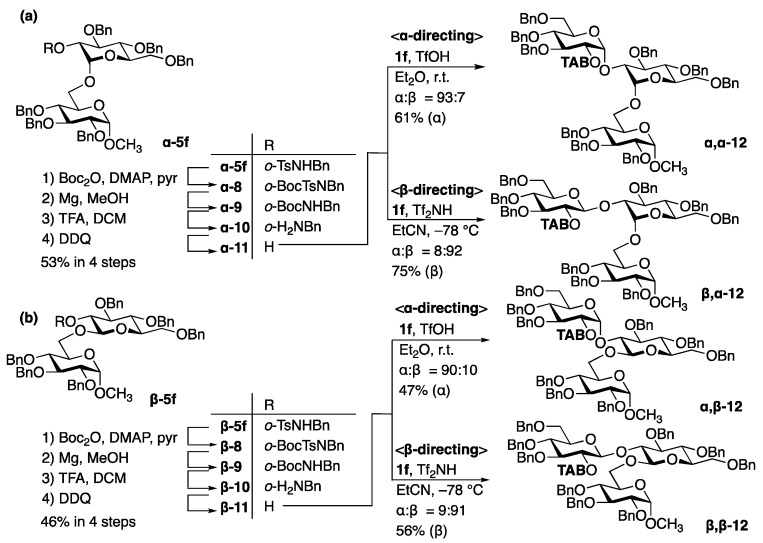
Synthesis of possible D-Glc-(1→2)-D-Glc-(1→6)-α-D-Glc-OMe derivatives using bimodal glycosylation methodology using TAB-protected donor. (**a**) Synthesis of α-D-Glc-(1→2)-α-D-Glc-(1→6)-α-D-Glc-OMe and β-D-Glc-(1→2)-α-D-Glc-(1→6)-α-D-Glc-OMe from **α-5f**; (**b**) synthesis of α-D-Glc-(1→2)-β-D-Glc-(1→6)-α-D-Glc-OMe and β-D-Glc-(1→2)-β-D-Glc-(1→6)-α-D-Glc-OMe from **β-5f**.

The construction of (1→2/3/4/6)-α-linkages of glucosides is a challenge because it is restricted by the 1,2-*cis*-stereocontrolled glycosylation methodologies and impacts assembly strategies [258]. The glucosyl donor equipped with a TAB group at the C2 position was examined for further elongation at that position after the deprotection of the TAB group of the glycosylation products to liberate the 2-hydroxy group [191]. The conversion was performed in four steps: (1) Boc protection, (2) deprotection of the Ts group via Mg treatment, (3) Boc deprotection, and (4) treatment with 2,3-dichloro-5,6-dicyano-1,4-benzoquinone (DDQ). Both α- and β-glycosides were converted into 2-hydroxy α- and β-D-Glc-(1→6)-α-D-Glc-OMe, respectively. (Scheme 4a,b). The resultant disaccharide acceptors were treated with C2-*o*-TAB-protected bimodal glucosyl donor under α- and β-directing conditions to afford four possible D-Glc-(1→2)-D-Glc-(1→6)-α-D-Glc-OMe derivatives (**12**), including α-D-Glc-(1→2)-α-D-Glc-(1→6)-α-D-Glc-OMe (**α,α-12**) [69,70,71,72,73]. This TAB approach should be applicable in the particular case of (1→2)-branched (1→3/4/6)-α-D-glycans or motifs.

**Scheme 5:**
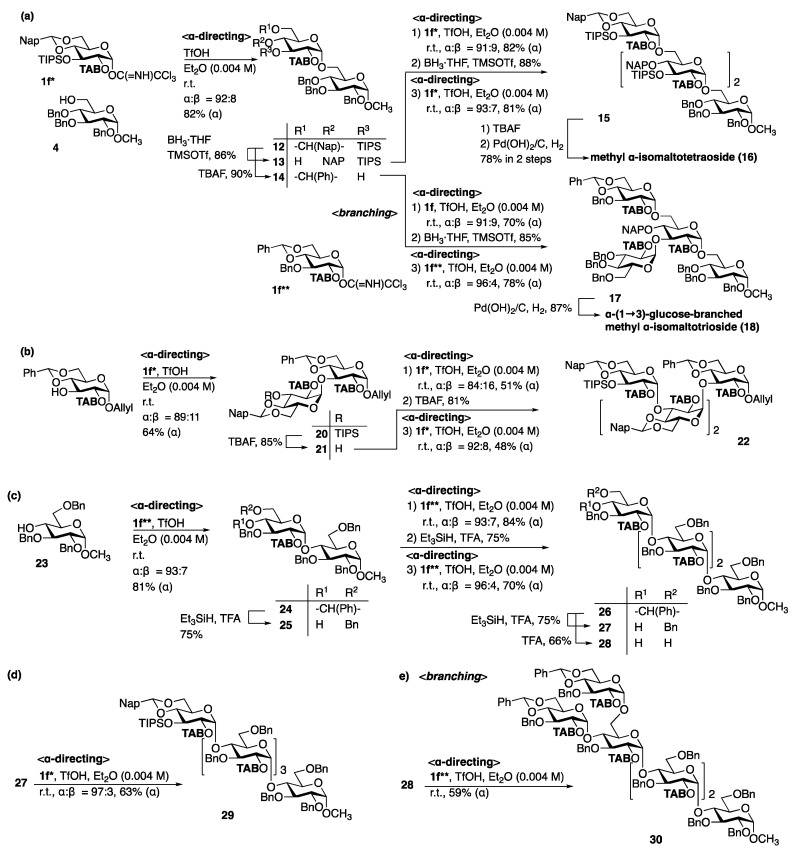
Synthesis of linear and branched α-glucan fragments using TAB-protected glucosyl donors. (**a**) Synthesis of linear (1→6)-α-D-glucan fragments and α-(1→3)-branched (1→6)-α-D-glucan fragment; (**b**) synthesis of linear (1→3)-α-D-glucan fragments; (**c**) synthesis of linear (1→4)-α-D-glucan fragments; (**d**) synthesis of linear (1→4)-α-D-glucan pentasaccharide; (**e**) synthesis of α-(1→6)-branched (1→4)-α-D-glucan hexasaccharide.

Figure 1 glucan fragment, a 6-hydroxy α-D-Glc-(1→6)-α-D-Glc-OMe derivative was prepared using 4,6-*O*-naphthylidene-3-*O*-triisopropylsilyl (TIPS)-2-*O*-(*o*-TAB)-D-glucopyranosyl trichloroacetimidate (1f*) and methyl 2,3,4-tri-*O*-benzyl-D-glucopyranoside (**4**) as the donor and acceptor, respectively (Scheme 5a) [193]. Several iterations of glycosylation under α-directing conditions and subsequent reductive ring-opening reactions to regioselectively liberate the 6-hydroxy group afforded methyl α-isomaltotetraoside [α-D-Glc-(1→6)]_4_-OMe (**16**).

The iterations of the glycosylation of allyl 4,6-*O*-benzylidene-2-*O*-(*o*-TAB)-D-glucopyranoside (**19**) with 4,6-*O*-naphthylidene-3-*O*-triisopropylsilyl-2-*O*-(*o*-TAB)-D-glucopyranosyl trichloroacetimidate (1f*) under α-directing conditions and subsequent deprotection of the TIPS group to liberate the 3-hydroxy group afforded the tetrasaccharide fragment (**22**) of linear (1→3)-α-D-glucan named pseudonigeran from *Aspergillus niger* (Scheme 5b).

The iterations of glycosylation of methyl 2,3,6-tri-*O*-benzyl-D-glcopyranoside (**23**) with 4,6-*O*-benzylidene-3-*O*-benzyl-2-*O*-(*o*-TAB)-D-glucopyranosyl trichloroacetimidate (1f**) was performed under α-directing conditions. Furthermore, the subsequent regioselective reductive ring-opening reaction to liberate the 4-hydroxy group afforded the pentasaccharide derivative (**26**) with the backbone structure of linear (1→4)-α-D-glucan (Scheme 5c), which was also elongated with 4,6-*O*-naphthylidene-3-*O*-triisopropylsilyl-2-*O*-(*o*-TAB)-D-glucopyranosyl trichloroacetimidate (1f*) under α-directing conditions to afford a hexasaccharide derivative (Scheme 5d).

#### 3.1.2. Bimodal Glycosylation Approach for the Synthesis of Branched α-glucans

For the construction of a branched α-glucan fragment, the α-(1→3)-branch in the linear (1→6)-α-D-glucan backbone, one of the components of dextran, was examined via the initial introduction of the α-(1→3)-branch (Scheme 5a, branching). The disaccharide obtained via the glycosylation of 4,6-*O*-naphthylidene-3-*O*-triisopropylsilyl-2-*O*-(*o*-TAB)-D-glucopyranosyl trichloroacetimidate (1f*) with methyl 2,3,4-tri-*O*-benzyl-D-glucopyranoside (**4**) under α-directing conditions, followed by the subsequent deprotection of the TIPS group, afforded the corresponding 3-OH disaccharyl acceptor (**14**). The subsequent α-selective glucosylation of the resultant acceptor with 3,4,6-tri-*O*-benzyl-2-*O*-(*o*-TAB)-D-glucopyranosyl trichloroacetimidate (**1f**) under α-directing conditions followed by a reductive ring-opening reaction to liberate the 6-hydroxy group and successive glycosylation under α-directing conditions afforded the tetrasaccharide fragment of a branched α-(1→6)-linked (1→3)-α-D-glucan (**18**) after hydrogenolysis.

The introduction of an α-(1→6)-branch into the linear (1→4)-α-D-glucan backbone was also examined via double α-glucosylation of the diol acceptor. After the synthesis of tetrasaccharide fragment (**26**) of linear (1→4)-α-D-glucan, deprotection of the benzylidene group by TFA in CH_2_Cl_2_ afforded the 4,6-diol (**24**) at the nonreducing D-glucose residue (Scheme 5c). The α-glucosylation of the resultant diol acceptor with 3 equiv. of the 4,6-*O*-benzylidene-3-*O*-benzyl-2-*O*-(*o*-TAB)-D-glucopyranosyl trichloroacetimidate donor (1f**) under α-directing conditions afforded a fully protected branched hexasaccharide fragment (**30**) in one pot (Scheme 5e, branching).

### 3.2. Application of ZnI_2_-mediated Stereoselective 1,2-cis α-glucosylation toward the Synthesis of α-glucans

#### 3.2.1. ZnI_2_-mediated Glycosylation Approach for the Synthesis of Linear α-glucans

**Scheme 6:**
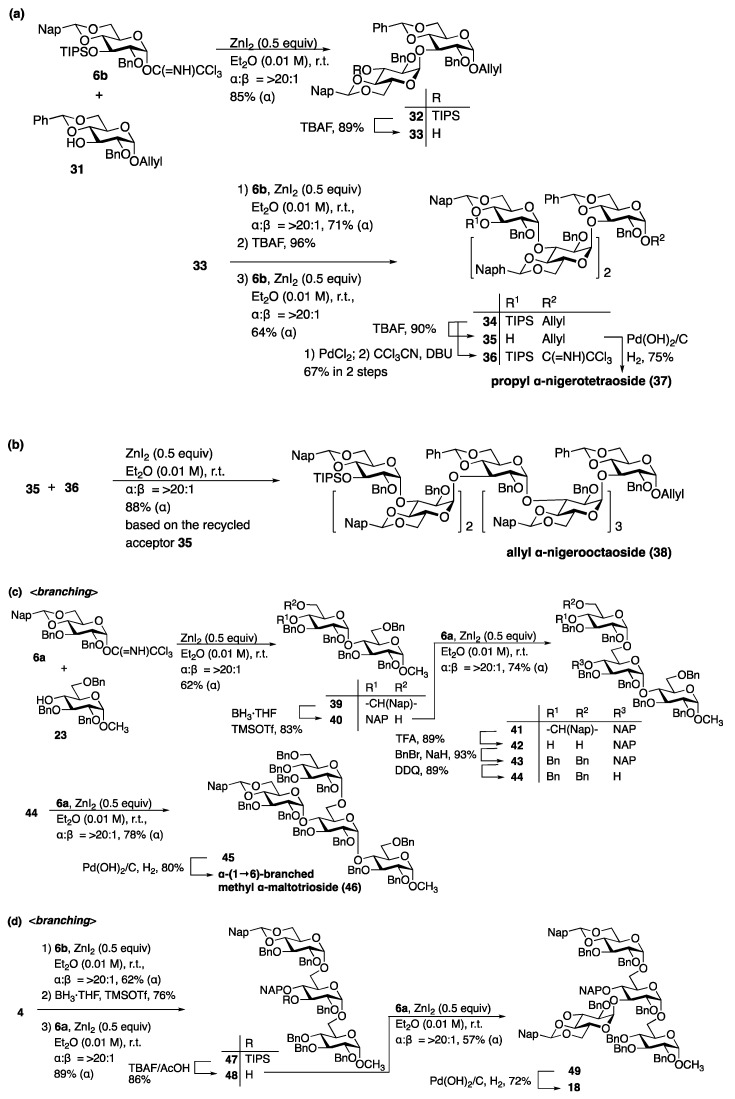
Synthesis of linear and branched α-glucan fragments using ZnI_2_-mediated 1,2-*cis* glycosylations. (**a**,**b**) Synthesis of linear (1→3)-α-glucan fragments, nigerotetraoside, and nigerooctaoside derivatives; (**c**) synthesis of α-(1→6)-branched (1→4)-α-glucantetrasaccharide; (**d**) synthesis of α-(1→3)-branched (1→6)-α-glucantetrasaccharide.

An alternative method for the synthesis of linear (1→3)-α-D-glucan, which constitutes the Pseudonigeran isolated from *Aspergillus niger*, has been shown using the ZnI_2_-directed α-glucosylation methodology [194]. After the first ZnI_2_-directed (1→3)-α-glucosylation between **6b** and **31**, the deprotection of the C-3-*O*-TIPS group of the resultant α-linked disaccharide (**32**) with TBAF in tetrahydrofuran (THF) afforded the corresponding disaccharide (**33**) with a C-3 hydroxy group (Scheme 6a). ZnI_2_-promoted glucosylation of the disaccharide acceptor (**33**) with the glucosyl donor (**6b**) gave the α,α-linked trisaccharide with high α-selectivity in a 71% yield. Repeating the deprotection and glucosylation steps yields the α,α,α-linked tetrasaccharide (**34**) stereoselectively, which was followed by the global deprotections via the desilylation and hydrogenolysis of **35** to complete the total synthesis of nigerotetraoside (**37**), a fragment of linear (1→3)-α-D-glucan. The desilylation of C-3-*O*-TIPS on tetrasaccharide (**34**) provided the acceptor (**35**), while the treatment of **34** with PdCl_2_ in methanol, followed by a reaction with CCl_3_CN and DBU, afforded the corresponding tetrasaccharyl trichloroacetimidate donor (**36**). Subsequent [4 + 4] coupling between the donor (**36**) and acceptor (**35**) with the ZnI_2_-promoted methodology under optimized conditions accomplished the synthesis of the target α-D-glucan nigerooctaoside (**38**) (Scheme 6b), suggesting the powerful synthetic applicability of the ZnI_2_-promoted glucosylation to oligosaccharide donors and acceptors with multiple repeating units (tetrasaccharides) in a fragment condensation strategy for assembling higher-molecular-weight glucans.

#### 3.2.2. ZnI_2_-mediated Glycosylation Approach for the Synthesis of Branched α-glucans

The versatility of the ZnI_2_-promoted glucosylation method has been shown by synthesizing branched α-glucan tetrasaccharides, such as (1→6)-α-branched (1→4)-α-D-glucan and (1→3)-α-branched (1→6)-α-D-glucan [194] (Scheme 6c,d).

The synthesis of α-(1→6)-branched (1→4)-α-D-glucan (**46**) was initiated by the stereoselective ZnI_2_-mediated glucosylation of the 4-OH acceptor **23** with 2-*O*-benzyl-4,6-*O*-benzylidene-3-*O*-2-naphthylmethyl (NAP)-D-glucopyranosyl donor (**6a**) for an α-(1→4)-linked disaccharide (**39**), followed by the liberation of C-6-OH using the selective reduction protocol of 4,6-*O*-benzylidene acetal with BH_3_·THF and trimethylsilyl trifluoromethanesulfonate (TMSOTf). The second ZnI_2_-mediated α-(1→6)-glucosylation of the resultant acceptor (**40**) with the donor (**6a**) provided the linear α-D-glucan trisaccharide fragment (**41**) stereoselectively. The subsequent hydrolysis of 4,6-*O*-benzylidene acetal using TFA in DCM afforded 4,6-diol (**42**), and a treatment of the resultant 4,6-diol of **42** with NaH and BnBr, followed by the selective removal of the NAP ether of **43** with DDQ, afforded the C-4-OH (**44**) at the residue in the middle of the same trisaccharide linkage (Scheme 6c). The resultant acceptor (**44**) was then glycosylated with a donor (**6a**) for branching under the ZnI_2_-mediated α-glucosylation conditions to provide the desired fully protected tetrasaccharide (**45**), which was followed by hydrogenolysis to complete the total synthesis of the branched α-glucan (**46**).

To introduce the (1→3)-branching to the (1→6)-α-D-glucan backbone, (1→6)-α-D-glucan trisaccharide derivative (**47**) was obtained by the stereoselective ZnI_2_-mediated glucosylation of the 6-OH acceptor (**4**) with donor (**6a**) followed by reductive ring-opening of naphthylidene acetal under BH_3_·THF and TMSOTf conditions, and the second stereoselective ZnI_2_-mediated glucosylation with the 3-*O*-TIPS-protected donor (**6b**). The liberation of the C-3-OH group by deprotection of the TIPS group of **47** with TBAF/AcOH afforded the corresponding **48**, and the third ZnI_2_-promoted α-glucosylation with the donor (**6a**) afforded the desired (1→3)-α-(1→6)-α-D-glucan tetrasaccharide (**18**) after hydrogenolysis via **49** with exclusive α-stereoselectivity (Scheme 6d). For the target branching structure, installing a functionality on the donor or acceptor moiety for the chemoselective liberation of the hydroxy group at a suitable position is required for the design of the synthesis, as shown here.

## 4. Conclusions

In this review, recent advances in stereoselective 1,2-*cis* glycosylation, focusing on α-glucosylation by bimodal glycosylation using *o*-TsNHbenzyl ether and ZnI_2_-mediated α-glucosylation, and their applications in the construction of various types of linear branched glycans, are discussed. These enable a systematic investigation of the glucan structure-biological activity relationships with a whole series of possible structural isomers that would become simpler and more facile. In addition, recent approaches toward cyclic α-glucans such as cyclodextrins with a small ring size (down to three glucose residues in the ring) used conformationally counterbalanced donors between equatorial- and axial-rich forms. The automated α-glucan synthesis of up to 20 glucose residues was reported by Yamada [259] and by Seeberger [260], respectively. As Yu reported very recently [199], the synthesis and structural analysis of α-glucans could be possible with MD calculations to allow a more reliable estimation of the van der Waals volumes of α-glucans. Further structural investigations are valuable and may enable various applications, such as biotechnology for medicine and cosmetics, functional foods, drug delivery, and immunological responses.

## Data Availability

Not applicable.
